# Development of gelatin based cryogel model for drug screening application in osteosarcoma

**DOI:** 10.1039/d6ra03165j

**Published:** 2026-07-03

**Authors:** Ponnamma Mandeda Madaiah, Rudra Nath Ghosh, Mathew Peter

**Affiliations:** a Manipal School of Life Sciences, Manipal Academy of Higher Education Manipal 576104 India; b Manipal Institute of Technology, Manipal Academy of Higher Education Manipal 576104 India mathew.peter@manipal.edu

## Abstract

Osteosarcoma remains a leading cause of primary bone cancer mortality due to its aggressive growth, early metastasis, frequent recurrence, and chemoresistance despite multimodal therapies. Conventional 2D cultures inadequately replicate the tumor microenvironment, limiting their predictive value for drug screening. Here, we developed a 3D gelatin-based cryogel platform (GelCryo) as a biomimetic platform for osteosarcoma drug screening; owing to their interconnected macroporosity, high permeability, tunable mechanics, and structural resilience, cryogels provide a major advance over conventional planar cultures to better mimic *in vivo* architecture, mechanics, and drug barriers and offer a more biomimetic environment for long-term cell growth and therapeutic testing. GelCryo scaffolds were fabricated at two glutaraldehyde crosslinking densities and systemic characterization revealed their macroporous structure (pore sizes 347–678 µm, porosity >93%) tunable stiffness (bulk modulus ∼14–16 MPa), high swelling capacity, and collagenase-responsive degradability, with the denser formulation exhibiting smaller pores, reduced swelling, and greater enzymatic stability. Both formulations supported sustained viability (>80% over 21 days), and proliferation (Ki67+ nuclei) of MG63 osteosarcoma cells over extended culture, confirming the cytocompatibility of the platform using alamarBlue assay. Critically, MTX dose–response revealed progressively increased IC_50_ values for MG-63 cells on 2D cultures (36.2 ± 11.1 µM), spheroids culture (69.7 ± 11.2), 1% GelCryo seeded scaffold (53.7 ± 12.9 µM) and 1.5% GelCryo seeded scaffold (99.8 ± 22.2 µM) suggesting chemoresistance in our 3D model. The 1.5% GelCryo scaffold exhibited the highest IC_50_ and recapitulating the cell–matrix mediated drug resistance typical of solid tumors. These findings establish GelCryo as a tunable biomaterial platform for osteosarcoma modeling, with strong potential to improve the physiological relevance of preclinical anticancer drug screening.

## Abstract 2

Osteosarcoma remains a leading cause of primary bone cancer mortality due to its aggressive growth, early metastasis, frequent recurrence, and chemoresistance despite multimodal therapies.

## Graphical abstract

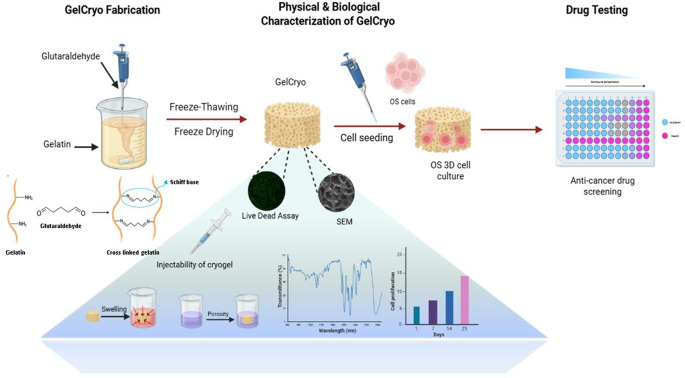

## 1. Introduction

Osteosarcoma (OS) stands as the leading primary malignant bone tumor, exhibiting peak prevalence among children and adolescents.^1^ Over the past few decades, chemotherapy and surgical treatments for OS have made significant advances, yet many patients are resistant to these treatments, eventually leading to surgical resection of the bone or it can lead to recurrence of the condition, ultimately developing into a metastatic disease.^2^ Despite standard treatment protocols, a substantial proportion of osteosarcoma cases exhibit resistance, contributing to persistently elevated mortality rates.^2^ This poor prognosis stems primarily from the disease's propensity for early metastasis to critical sites like the lungs, which markedly heightens comorbidity risks and fatal outcomes. Secondly, early detection of these cancer cells is difficult due to the lack of specific markers.^3,4^

These ongoing clinical challenges arise from multiple factors, including the inherently aggressive nature of the tumor, its pronounced tendency to metastasize, and, importantly, the development of resistance to standard chemotherapy treatments. It is therefore essential to develop novel therapeutic approaches, as well as predictably preclinical models, to facilitate clinical translation of experimental findings.^5^ As a result of our limited knowledge on OS, research on new treatments is hindered due to the lack of accurate *in vitro* models that reproduce OS signalling and drug responses.^6^

The traditional 2D cell culture models of tumours are not capable of accurately mimicking the complex physiological and pharmacological responses at organ-level, despite being convenient, cost efficient along with high-throughput capability.^7^ Due to the lack of interaction between cells and matrix, they do not accurately reflect the *in vivo* tumor microenvironment for drug testing.^8^ A cancerous tumour is composed of a wider range of heterogeneous tissue-specific cell types that are better able to form cellular connections and interact with one another. Unlike cells maintained in traditional 2D monolayers, three-dimensional (3D) culture systems faithfully replicate the architectural intricacy of native tumor microenvironments, enabling more precise recapitulation of physiological tumor behaviors and responses.^9^ A 3D cell culture model represents the structural and functional complexities of *in vivo* tissues, so that it can be used to study dynamic processes such as tumor development.^10^ The transition to 3D cell culture represents a major advancement offering a more physiologically relevant model for studying cellular processes and disease. Despite some challenges, the benefits of 3D cell culture outweigh the limitations of traditional 2D culture.^10^

In 3D cell cultures, scaffolds play a vital role in enabling cells to interact, grow, and proliferate, thereby enhancing cell survival and function. Scaffold-supported three-dimensional (3D) cell culture models have emerged as a critical platform in oncology research, providing a physiologically relevant environment to investigate tumor dynamics, therapeutic responses, and reciprocal interactions between neoplastic cells and their stromal niche.^6^ Although there are various types of scaffolds for 3D cell culture, in recent years, cryogels have gained immense popularity as in the field of tissue engineering due to its ability to mimic the extracellular matrix.

Cryogels consist of highly porous hydrogel structures known for their outstanding biocompatibility, mechanical and thermal strength, biodegradability, micro-scale interconnected porosity, and chemical crosslinking, which makes them more advantageous compared to other biomaterials, especially for biomedical applications. A key advantage to them is that the solvent remains unfrozen during the freeze-thawing process, which produces porogens as a result.^11^ Eventually, the porogen forms a macroporous structure that is elastic, stable and interconnected. On the other hand, other biopolymers are synthesized by the process of freeze-drying which is more time-consuming and yields less porous structures. Cryogels overcome hydrogels shortcomings in mechanical robustness, storage convenience, handling ease, and sterilization efficiency.^12,13^ Due to the biodegradable and biocompatible nature, gelatin is considered as a promising biomaterial for drug delivery and tissue engineering.^14^ Several peptide sequences found in gelatin promote cell adhesion and enzyme degradation.^15^ As a result, gelatin cryogels serve as efficient scaffolds for cell adhesion and proliferation, thereby aiding in drug testing. Due to the presence of amino acids in gelatin (COOH, NH_2_), it can be dissolved in water at high temperatures and is thermally reversible in a gel below 40 °C.^16^ Owing to its retention of collagen-like bioactive motifs and the formation of a highly interconnected macroporous architecture, cryogels made from gelatin can closely recapitulates the structural organization and transport properties of the osteosarcoma ECM than two-dimensional monolayers or spheroids models. Cryogels made from gelatin can also have another application in the field of tissue engineering (especially in drug delivery, wound healing, and cell regeneration studies) due to their biodegradable and biocompatible nature.^17^

In this study, we aim to develop a gelatin-based 3D cryogel culture model of osteosarcoma to accurately replicate the tumour architecture and cellular interactions observed *in vivo*. Using scaffold-based techniques, we generate a 3D model that enables the assessment of drug efficacy under physiologically relevant conditions. This model offer valuable insights into the drug response in osteosarcoma and can function as a platform for screening new therapies, ultimately aiding in the development of more effective treatments for osteosarcoma patients.

## 2. Materials and methods

### 2.1. Materials

Gelatin powder (Loba Chemie Pvt. Ltd, India), sodium borohydride (NaBH_4_), sodium hydroxide (NaOH), ethanol (99.9% laboratory grade), and potassium bromide (KBr) were sourced from Loba Chemicals, India. Cell culture reagents, including Dulbecco's Modified Eagle Medium (DMEM) with high glucose, DMEM without sodium bicarbonate and phenol red, Dulbecco's Phosphate Buffered Saline (PBS), fetal bovine serum (FBS), antibiotic solution (100X) 10,000U Penicillin and 10 mg Streptomycin per mL in 0.9% saline, and 250 µg mL^−1^ amphotericin-B solution, were acquired from Himedia, India. Additional reagents such as Trypsin-EDTA Solution 1X, 4% paraformaldehyde, Triton X-100, and bovine serum albumin (BSA) were also obtained from Himedia. Alamar Blue (Resazurin), calcein AM, and propidium iodide (PI) were purchased from Invitrogen Thermo Fisher Scientific, India. Collagenase type II and lysozyme were supplied by Himedia, and the bicinchoninic acid (BCA) assay kit was from Sigma-Aldrich, USA. Methotrexate (MTX, C_20_H_22_N_8_O_5_, Molecular weight = 454.45 g mol^−1^) was provided by Yarrow Chem Products. The HiPurA Multi-Sample DNA Purification Kit was obtained from HiGenoMB, Himedia.

### 2.2. Fabrication of gelatin cryogel (GelCryo)

#### 2.2.1. GelCryo preparation

The synthesis of gelatin cryogels (GelCryo) was carried out using both freeze-thaw and freeze-drying protocol. Two concentrations of glutaraldehyde (GA) (1% and 1.5% v/v) were used as crosslinkers, using a constant weight/volume (w/v) % of gelatin. 2.5 g of gelatin was first dissolved in 50 mL distilled water at 40–50 °C under continuous stirring conditions on a magnetic stirrer till gelatin dissolves completely. Once the mixture is cooled to 30 °C, add 1 mL of the prepared solution to each well of the 24-well plate and 1 mL of chilled glutaraldehyde of appropriate percentage. For cell culture, thin GelCryo is prepared by combining 7 mL gelatin solution and 7 mL of chilled glutaraldehyde of appropriate percentage in 15 mL Petri plates lined with parafilm. The mixture was then incubated overnight at −20 °C to promote crosslinking. Following cryogelation, the molds were subjected to a thaw cycle in distilled water for 2 h, followed by freezing for 4 h at −20 °C. The cryogels were subjected to three free-thaw cycles. Post-treatment the gels were soaked in freshly prepared 1% NaBH_4_ for 1 h, followed by washing with distilled water to remove excess unreacted glutaraldehyde. The samples were then frozen at −20 °C overnight and lyophilized for over 24 h. Post-drying thin GelCryo scaffolds for cell culture applications are punched using 8 mm biopsy punches and were sterilized by autoclaving at 121 °C and 15 psi for 15 minutes, followed by UV exposure for 30 minutes.

#### 2.2.2. Optimization of GelCryo

To optimize cryogel formation, various concentrations of gelatin (1%, 3%, 5% w/v) and glutaraldehyde (0.2%, 0.4%, 1.0%, 1.5% v/v of 25% stock solution) were systematically evaluated (Fig. S1). Each combination was subjected to the cryogelation process. At low gelatin concentration (1%), all glutaraldehyde levels yielded hydrogels or hydrogel-like materials lacking the desired mechanical strength and porosity. At 3% gelatin, increasing glutaraldehyde concentrations led to weak cryogel formation. At 5% gelatin, higher glutaraldehyde levels (1.0%, 1.5%) produced robust cryogels with suitable mechanical and structural properties, as summarised in the optimisation matrix.

### 2.3. Characterization of GelCryo

#### 2.3.1. FT-IR spectroscopy

Fourier-transform infrared (FT-IR) spectra for gelatin and GelCryo samples were recorded to examine the chemical functional groups as well as their interactions with gelatin and crosslinking agents using Shimadzu IR Spirit spectrometer. Dried samples were mixed with KBr in a 1 : 6 ratio and pressed into pellets for analysis over the 400–4000 cm^−1^ range.

#### 2.3.2. SEM analysis

Scaffold architecture was analyzed using scanning electron microscope to understand the size and morphology of GelCryo. Crosslinked GelCryo were prepared for SEM using the above-mentioned protocol. Before imaging, both concentrations thin sections of the samples mounted on aluminum stubs, were sputter-coated with gold and imaged by using Zeiss EVO19 MA18. Pore sizes were quantified using ImageJ software by measuring 100 pores per sample.

#### 2.3.3. Porosity measurement

Porosity was determined using the liquid displacement method with *n*-hexane as the displacement fluid.^18^ The dried scaffold was weighed, submerged in *n*-hexane, and the change in weight was used to calculate porosity using the formula: 
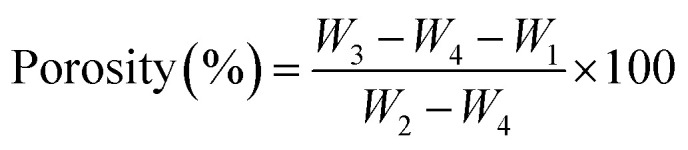
where *W*_1_ is the dry scaffold weight, *W*_2_ is the container with *n*-hexane, *W*_3_ is the container with scaffold and *n*-hexane, and *W*_4_ is the container with remaining *n*-hexane after scaffold removal. Experiments were performed in duplicate with triplicates for each sample.

#### 2.3.4. Mechanical testing

Mechanical strength of the dried cryogel samples were assessed using a universal testing machine (UTM, Shenzhen Wance, India). Cryogels were sliced into uniform parallel discs with a specific diameter and placed on a stub. Testing was performed by compressing the cryogel discs at 50 *N* load at a rate of 10 mm min^−1^ displacement rate. The bulk modulus of the GelCryo was calculated by plotting a graph of stress (kPa) *versus* strain(%). The slope of the linear 5–10% region of the stress/strain curves, calculated as: 
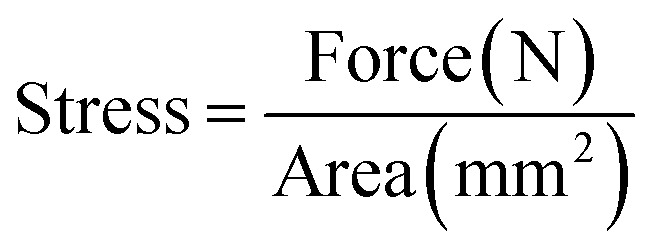

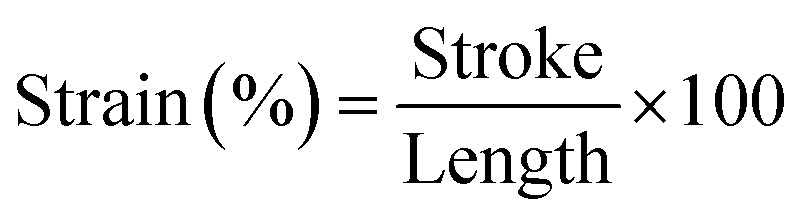


#### 2.3.5. Swelling studies

The weights of the dried GelCryo samples were measured (*W*_d_). The dried cryogel samples were incubated in 0.1 M PBS at 37 °C, and periodically weighed (*W*_t_) to determine swelling percentage: 
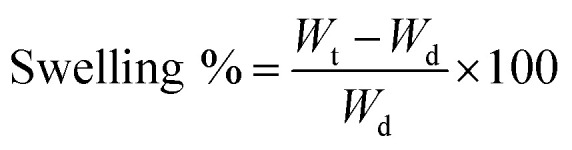


#### 2.3.6. *In vitro* degradation

Cell-free *in vitro* degradation rates were determined by monitoring the weight loss of the GelCryo scaffolds at designated time points. The cryogels were incubated in 0.1 M PBS with 0.1% sodium azide at 37 °C for 12 h or until they reached equilibrium. The weight of the swollen cryogels was noted as the initial weight (*W*_0_). To study *in vitro* degradation, the swollen GelCryo scaffolds were transferred into a Petri dish containing collagenase type II solution of 2.5 U mL^−1^ and 25 U mL^−1^ concentrations prepared in PBS (pH 7.4) and periodic weights (*W*_t_) were recorded at different time points. To determine the degradation percentage of the cryogel the following equation was used:
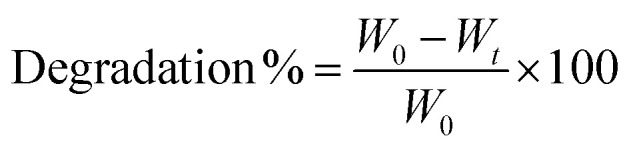
Mass Loss % = 100 − Degradation %

#### 2.3.7. Protein adsorption

GelCryo prepared in 24-well plates were incubated 10% FBS or 1% BSA dissolved in PBS in v/v % in shaking incubator for a time period of 1 and 6 h at 37 °C. Post the specified incubation the cryogels were transferred to fresh well, following which it is given PBS wash thrice. Adsorbed proteins were eluted by soaking the cryogels in elution buffer (0.5% Chaps, 10% SDS) overnight and concentrations were quantified using the BCA assay.^19^ Absorbance at 562 nm was measured using a microplate reader (BioTek Synergy H1 reader), and protein concentration was determined using a standard curve.

#### 2.3.8. Water contact angle

Contact angles of gelatin films with varying glutaraldehyde concentrations were measured using a contact angle meter (HOLMARC, INDIA). Water droplets were placed on the gelatin coated film surface, and angles were analyzed using ImageJ. Measurements were repeated at three locations per sample.

### 2.4. Biocompatibility assessment

#### 2.4.1. Cell culture

NIH/3T3 (mouse fibroblast) and MG63 (human osteosarcoma) cell lines were obtained from NCCS, Pune. Both the cells were cultured in complete DMEM media supplemented with 10% FBS and 1% antibiotic solution (10 000 U Penicillin and 10 mg Streptomycin) (HIMEDIA). For scaffold cultures, 1% amphotericin-B was also added. Cells were cultured in a humidified 5% CO_2_ incubator at 37 °C and cell culture medium was replaced every 48 h and sub cultured when required 80% confluency was reached. Prior to *in vitro* studies, cells were trypsinized with 0.25% Trypsin and 0.001% EDTA (HIMEDIA), collected *via* centrifugation (1500 rpm for 5 min) and resuspended in fresh media. Trypan blue exclusion was performed to count the cells, following which the cells are resuspended with the required density for cell seeding along with determining cell viability prior to seeding.

#### 2.4.2. 3D Culture: scaffold seeding

Sterilized 8 mm cryogel scaffolds were pre-incubated in 500 µl supplemented DMEM media for 1 h. 2 × 10^5^ cells were seeded per scaffold and incubated for 2 h before adding an additional 1 mL DMEM media to allow adhesion of the cells to the cryogel. Media was replaced every 48–72 h for long-term culture.

#### 2.4.3. 3D Spheroid culture

Spheroids were generated using both the liquid displacement and hanging drop methods. For the liquid displacement method, U-shaped agarose-coated wells were prepared, and MG63 cells were seeded and incubated with DMEM media to form spheroids. The hanging drop method involved suspending 1 × 10^3^ cells in 30 µl of supplemented DMEM media containing 1.25% carboxymethyl cellulose (CMC) (w/v) droplets on a culture dish lid, inverted over a base with media. After 5 days, spheroids were transferred to U-shaped agarose-coated wells containing supplemented DMEM media for further experiments. The hanging drop method produced more uniform spheroids and was used for subsequent studies.

#### 2.4.4. 3D Culture cell viability assay

The trypan blue exclusion method was used to assess scaffold viability in 3D culture. Cell viability in scaffolds was evaluated at 48 h, 7, 14, and 21 days using trypan blue exclusion. Each scaffold was physically disrupted with a blunted needle before being treated with 0.5 mL of 0.25% Trypsin at 37 °C for 10 minutes to detach cells from the scaffold. After 10 min, 0.5 mL of DMEM media was added to neutralise and inhibit trypsin activity. The 1 mL of the scaffold supernatant was added to a 1.5 mL microcentrifuge tube and centrifuged at 1500 rpm for 5 min to pellet the cells. The supernatant was removed, and the cell pellet was resuspended in just 20 µl of DMEM media. A hemocytometer was filled with 10 µl of cells per trypan blue suspension, and cells were counted in the four corners. Therefore, cells were released by mechanical and enzymatic dissociation, counted with a hemocytometer, and viability was calculated as:
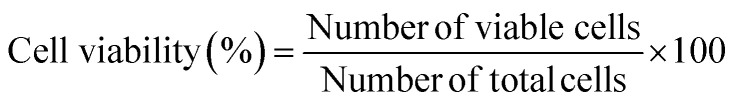


#### 2.4.5. Cell proliferation assay

Cell proliferation was assessed using the Alamar Blue assay. Scaffolds were seeded with 1 × 10^5^ cells, incubated and metabolic activity measured at days 1, 7, 14, 21, and 28. Controls included scaffolds with media only. Media was changed every other day. Alamar Blue 10% working solution was prepared by mixing 1 mL Alamar Blue stock solution with 9 mL HBSS (without phenol red and serum) and filtered using sterilized 0.2 µm filter and wrapped in foil until further use. The sterile test scaffolds were transferred to 24 well tissue culture plates along with 1 mL complete cell culture media. The media was discarded and given a PBS wash before adding 500 µl of Alamar Blue to each well and incubated at 37 °C for 4 h. Alamar Blue (150 µl) working solution (post-incubation with scaffolds) was transferred to a 96-microtiter black well plate and fluorescence was measured at 570 nm (Ex 560 nm/Em 590 nm). The seeding density and media volume was kept constant in both cryogel group and in control group.

#### 2.4.6. Live/dead staining

After the incubation for predetermined days, viability of the cells in the GelCryo scaffold was determined by LIVE/DEAD^®^ viability assay and cell proliferation assay. Briefly after the cell culture medium was removed from each scaffold, 500 µl of DMEM containing 2 µM Calcein AM and 2 µM propidium iodide (PI) was added to stain live and dead cells, respectively. After incubation for 1 h at 37 °C and 5% CO_2_, the scaffolds were washed with PBS. Following this, the scaffolds were then transferred to a confocal dish and examined *via* fluorescence microscopy (Olympus IX73 inverted microscope).

#### 2.4.7. Ki 67-DAPI staining

The staining solution was prepared by adding Ki-67 stock to PBS to achieve 1 : 100 dilution. To each scaffold, the staining solution was added to the treated and untreated scaffolds with cells, and the scaffolds were incubated for 24 h at 4 °C, followed by DAPI staining for 30 minutes. The excess stain was removed by a couple of PBS washes. The samples were imaged using fluorescence microscopy (Olympus IX73 inverted microscope). The entire procedure was carried out in the dark.

### 2.5. Drug testing

#### 2.5.1. Dose–response and IC_50_ calculation

To assess cytotoxic response to chemotherapeutic drug, methotrexate (MTX), the viability of the cells Alamar Blue assay. MTX stock solution (1000 µM) was prepared in 0.5% DMSO and 0.1 M NaOH. For 2D studies, 5 × 10^3^ cells were seeded in 96-well plates and incubated overnight. After 24 h the cells were treated with serial MTX concentrations (0.01, 0.1, 1, 10, 100 µM). After 48 h of drug incubation, cell viability was measured using alamarBlue. Dose–response curves were generated, and IC_50_ values were calculated using a four-parameter logistic model. MG63 spheroids, prepared by the hanging drop method from 1 × 10^3^ cells per spheroid, as well as scaffold-seeded MG63 cells, NIH3T3 cells, and co-culture samples, were analyzed using the same protocol. In brief, 2 × 10^5^ cells were seeded onto each scaffold and incubated for 2 h prior to the addition of fresh medium. Following complete cellular colonization of the scaffolds, the cultures were treated with serial concentrations of methotrexate (0.01, 0.1, 1, 10, and 100 µM) for 48 h, after which cell viability was quantified using the Alamar Blue assay.

#### 2.5.2. Drug retention and 2D IC_50_ dose cytotoxicity evaluation

Methotrexate (MTX) at a concentration of 100 µM was prepared in PBS and UV-visible spectroscopy at 303 nm was performed to estimate the initial absorbance of MTX drug. Subsequently, a 4 mm × 1 mm (diameter × height) GelCryo scaffold was immersed in 1 ml of 100 µM MTX solution and incubated at 37 °C under static conditions for 24 hours. After incubation, the GelCryo scaffold was taken out and squeezed to release the trapped solution in the GelCryo matrix and analysed for its absorbance at 303 nm. The drug retention percentage was calculated by the formula:
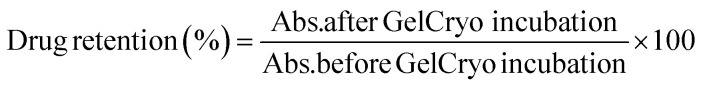


#### 2.5.3. Zeta potential analysis of GelCryo and MTX-GelCryo scaffolds

The zeta potential of GelCryo scaffolds before and after methotrexate (MTX) adsorption was measured to assess changes in the scaffold's surface charge. Briefly, GelCryo scaffolds and GelCryo scaffolds incubated with 100 µM MTX for 24 h were finely ground and dispersed in phosphate-buffered saline (PBS, pH 7.0). The resulting suspensions were analyzed using a Zetasizer Lab Pro (Malvern Panalytical, UK) at 25 °C. Measurements were performed using a dispersant refractive index of 1.33 and a dispersant viscosity of 0.88 cP. Three independent measurements were performed for each group, and the mean zeta potential was recorded. The obtained zeta potential values were compared to evaluate the effect of MTX adsorption on the electrokinetic properties of the GelCryo scaffold and to investigate potential scaffold-drug interactions.

#### 2.5.4. Conditioned medium assay for evaluating methotrexate sequestration by GelCryo scaffold

To assess whether the GelCryo Scaffolds altered methotrexate availability, a conditioned medium assay was performed. Sterile scaffolds were placed in 24-well plates and incubated with 1 mL of complete culture medium containing methotrexate at the IC_50_ determined in 2D culture (36 µM). The scaffolds were maintained at 37 °C in a humidified atmosphere containing 5% CO_2_ for 24 h. Following incubation, the supernatant was carefully collected and centrifuged at 1000×*g* for 5 min to remove any suspended particles. The recovered medium, referred to as scaffold-conditioned drug medium, was subsequently applied to cells cultured in conventional 2D monolayers. After the designated treatment period, cell viability was quantified using a resazurin-based fluorescence assay. All experiments were performed in triplicate, and results are presented as mean ± standard deviation.
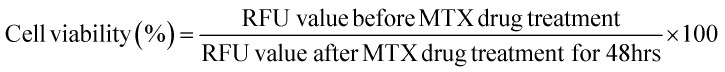


#### 2.5.5. Statistical analysis

All experiments were performed in triplicate and repeated three times. Data are presented as mean ± SD. Statistical analysis was conducted using one-way ANOVA, with significance set at *p < 0.05.

## 3. Results

### 3.1. Characterisation of GelCryo

#### 3.1.1 FTIR

**Fig. 1:**
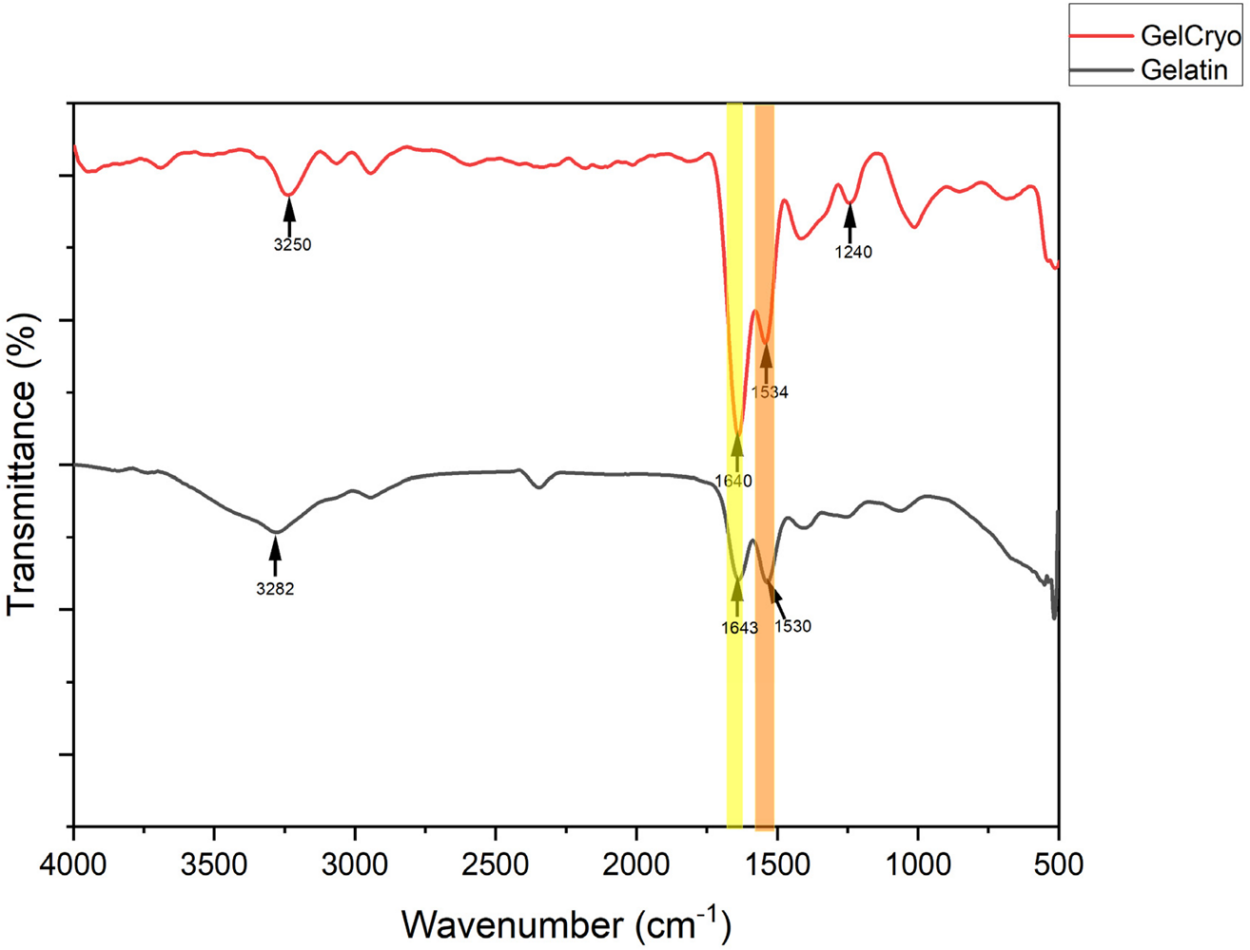
FTIR spectrum of GelCryo and gelatin.

FT-IR spectroscopy was done to determine the interatomic and molecular bonding present between the functional groups of the gelatin in GelCryo. FTIR spectroscopy analysis of GelCryo revealed a pronounced absorption band at 1640 cm^−1^, corresponding to the stretching of carbonyl (C=O) groups. This spectral feature supports successful crosslinking of gelatin by glutaraldehyde, consistent with previous findings reported by Lee *et al.*,^20^ The peak at 1530 cm^−1^ corresponds to the amide II region, which is mainly associated with N–H bending motions within the gelatin structure. A broad absorption band near 3300 cm^−1^, attributed to N–H stretching vibrations. This attenuation aligns with the consumption of free amino groups during crosslinking reactions. As observed in previous studies^21,22^ the preserved amide III band (∼1240 cm^−1^) in both spectra indicates maintenance of the collagen-like triple helix structure, essential for cell adhesion and proliferation in biomedical scaffolds (Fig. 1).

#### 3.1.2 Tunable porosity of GelCryo scaffolds through crosslinking control

**Fig. 2:**
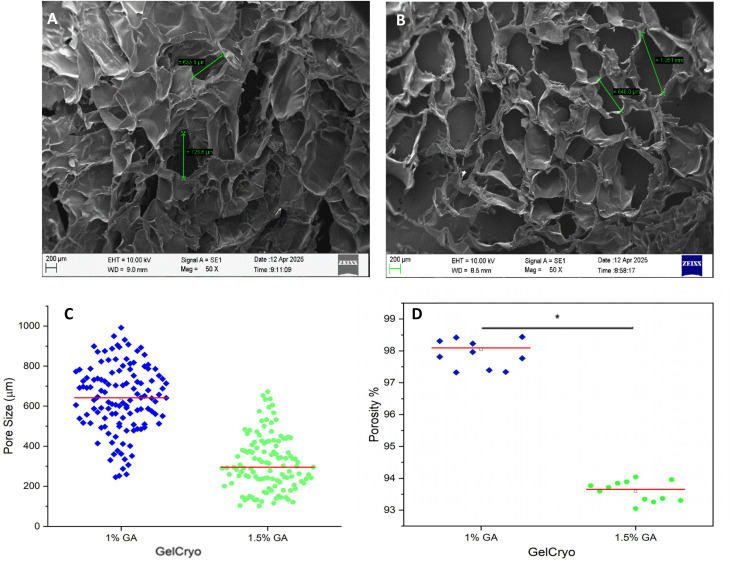
SEM images of transverse cross sections across all concentrations of glutaraldehyde confirmed the porous structure of the GelCryo (A) SEM image of 1% glutaraldehyde (GA) GelCryo (B) SEM image of 1.5% GA GelCryo (C) pore size of 130 pores of each sample (D) porosity of GelCryo of various glutaraldehyde concentrations by liquid displacement method. Statistical significance was tested with one-way ANOVA (*p < 0.05). (Magnification 50×, scale bar-200 µm).

Scanning electron microscopy (SEM) analysis confirmed that glutaraldehyde concentration significantly influenced GelCryo architecture (Fig. 2A and B). The 1% GA scaffold displayed a highly porous, interconnected network with an average pore size of 678.31 ± 212.92 µm, while increasing GA to 1.5% reduced the average pore size to 347.28 ± 194.96 µm, yielding a denser framework (Fig. 2C). Porosity analysis by liquid displacement further demonstrated a decline from 98.03% at 1% GA to 93.59% at 1.5% GA (Fig. 2D).

This inverse relationship between crosslinking density and pore formation is consistent with prior reports showing that restricted ice crystal growth at higher GA concentrations resulted in finer pores and lower porosity.^23^ The porosity, pore size, and the overall arrangement of these pores play crucial roles in enabling cells to penetrate the biomaterial framework and support subsequent tissue development.^24^ Functionally, pore sizes within the 200–500 µm window are optimal for nutrient diffusion, cell infiltration, and osteogenic response.^25,26^ The interconnected pores of the scaffolds are also responsible for the glycosaminoglycan (GAG) secretion with enhanced cell viability and proliferation.^27^ Thus, the 1% GA scaffold favours proliferation through larger interconnected pores, whereas 1.5% GA provides a denser network supportive of early differentiation, highlighting tunability of GelCryo for osteogenic tissue modelling.

#### 3.1.3. Crosslinking density influences the swelling behavior and mechanical stiffness of GelCryo scaffold

**Fig. 3:**
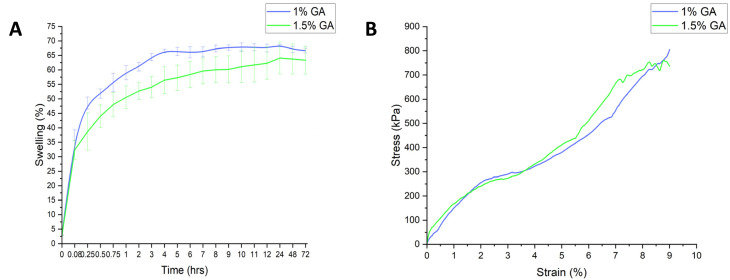
Swelling behavior of GelCryo samples (A) swelling percentages of GelCryo with different concentrations of glutaraldehyde (GA) equilibrated in PBS at 37 °C within a shaking incubator during 72 h incubation period (*n* = 3) mean ± sd (B) stress–strain curve was obtained through initial compression of GelCryo until 10% strain.

Swelling analysis in PBS demonstrated that GelCryo scaffolds rapidly reached equilibrium within 10 minutes, with dimensions remaining stable thereafter (Fig. 3A). The 1% GA scaffold exhibited significantly higher swelling compared to 1.5% GA, consistent with its larger pore size and lower crosslinking density. In contrast, the denser 1.5% GA network limited water uptake, correlating with its higher bulk modulus. This inverse relationship between crosslinking and swelling behavior agrees with established models where increased crosslinking restricts matrix hydration.^28^ Functionally, scaffolds with higher swelling and porosity enhance effective nutrient and oxygen diffusion, thereby promoting cell infiltration and metabolic activity, which is critical for sustaining viability in 3D cultures.^29^

Mechanical testing revealed that increasing glutaraldehyde concentration enhanced scaffold stiffness (Fig. 3B). GelCryo crosslinked with 1.5% GA exhibited a higher bulk modulus (15 857 ± 10.35 kPa) compared to 1% GA scaffolds (13 696 ± 2.72 kPa), indicating reduced compressibility and improved resistance to deformation. This reinforcement arises from higher crosslinking density, which strengthens the polymeric network. Enhanced mechanical robustness is critical for bone tissue applications, where scaffolds must withstand physiological loads while maintaining structural stability, thereby supporting essential cellular processes such as adhesion, migration, and osteogenic differentiation.^30^

#### 3.1.4 Crosslinking modulates collagenase-induced degradation of GelCryo cryogels

**Fig. 4:**
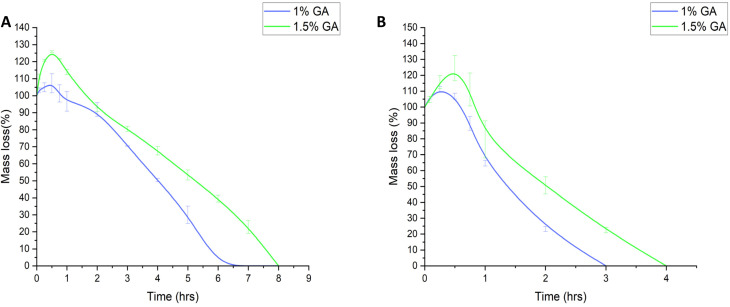
*In vitro* degradation profile of GelCryo in collagenase solution at varying concentrations respectively. Percentage of mass loss of the GelCryo in presence of (A) collagenase (2.5 U ml^−1^), (B) collagenase (25 U ml^−1^) during 24 h incubation at 37 °C in shaking incubator. Data represented as mean ± SE of mean, (*n* = 3).

Enzymatic degradation profiles of GelCryo scaffolds revealed the enzyme-mediated (biological) stability of the gelatin cryogels, demonstrating a clear influence of crosslinking density and collagenase concentration (Fig. 4). At 2.5 U ml^−1^ collagenase, 1% GA scaffolds completely degraded within 6–7 h, whereas 1.5% GA required 8–9 h. At the higher concentration (25 U ml^−1^), both scaffolds degraded rapidly, with 1% GA breaking down within 3–4 h and 1.5% GA by ∼4 h. The observed sigmoidal degradation pattern, characterized by a lag phase followed by rapid mass loss, is typical of crosslinked protein networks, where partial cleavage increases enzyme accessibility, accelerating subsequent breakdown.

Higher resistance in 1.5% GA scaffolds reflects their denser network, which reduces enzymatic access to cleavage sites, consistent with crosslink density-dependent stability.^31^ Tuneable degradation offers advantages for tailoring scaffold lifetime to experimental demands. Notably, gradual breakdown at physiological enzyme levels supports applications in long-term tissue culture and controlled drug release, while accelerated degradation under higher enzymatic activity mimics remodelling in inflammatory environments.^32^

The prolonged degradation profile under lower enzyme conditions suggests the potential for controlled drug release. Sigmoidal degradation kinetics are characteristic of crosslinked protein networks, where initial degradation events create additional enzyme binding sites, leading to autocatalytic degradation processes.^33^ The faster degradation rates observed at higher collagenase concentrations (25 U ml^−1^) simulate conditions that might be encountered in inflammatory environments or during active tissue remodelling due to the upregulation of metalloproteinases (MMP).^32^ Under these conditions, even the more heavily crosslinked 1.5% GA samples degraded rapidly, suggesting that the scaffolds would respond appropriately to elevated enzymatic conditions *in vivo*.

#### 3.1.5 Crosslinking-dependent modulation of hydrophobicity and protein adsorption in GelCryo scaffolds

**Fig. 5:**
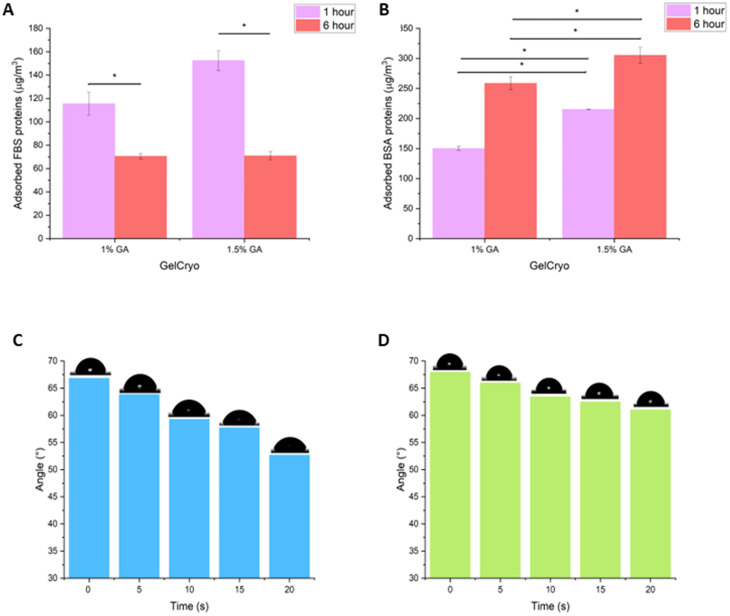
BSA and FBS protein adsorption. (A) BSA protein adsorption on the GelCryo of varying glutaraldehyde concentrations normalized to the volume of the scaffolds (B) FBS protein adsorption on the GelCryo of varying glutaraldehyde concentrations normalized to the volume of the scaffolds. Water contact angle of the 3D GelCryo (C) 1% GA GelCryo (D) 1.5% GA GelCryo. Statistical significance was tested with one-way ANOVA (*p < 0.05), (*n* = 2).

Protein adsorption assays revealed distinct trends for single-protein (BSA) *versus* serum protein mixtures (FBS) (Fig. 5). Both 1% and 1.5% GA GelCryo scaffolds showed time-dependent increases in BSA adsorption, with the 1.5% GA sample exceeding 300 µg µm^−3^ at 6 h, indicating that higher crosslinking and surface hydrophobicity favour sustained BSA retention. In contrast, FBS adsorption was initially higher but declined significantly by 6 h for both scaffolds, converging to comparable levels. This transient behaviour reflects competitive binding and desorption of diverse serum proteins, which equilibrate more rapidly on biomaterial surfaces.

Water contact angle (WCA) measurements supported these trends. The 1% GA GelCryo showed a progressive decrease from 69° to 54° within 20 s, whereas the 1.5% GA sample retained hydrophobicity more effectively, stabilising at 62°. This higher hydrophobic stability is directly correlated with enhanced BSA adsorption. Since protein adsorption governs presentation of adhesive motifs such as RGD peptides,^34^ tuning crosslinking allows modulation of scaffold bioactivity—favouring either stable protein retention for tissue engineering,^35^ or dynamic exchange for rapid remodelling^36^ (Fig. 5).

## 4. Biocompatibility assessment

### 4.1 3D cell viability assay

**Fig. 6:**
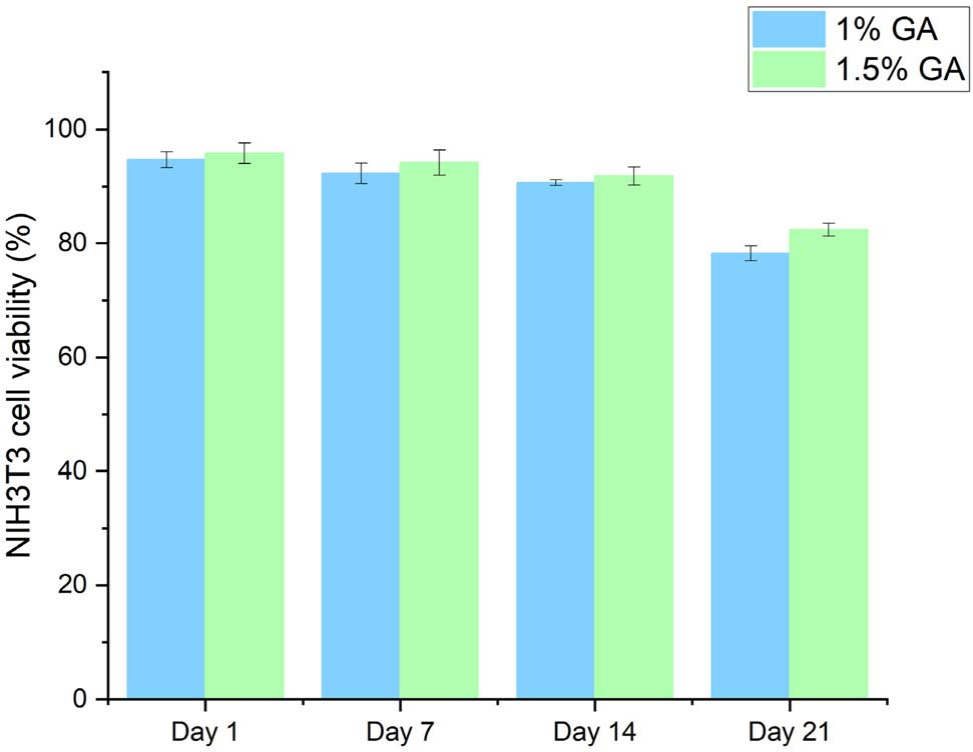
Trypan blue exclusion was used to assess cell viability in 3D GelCryo. Viability remained constant up to 14 days. Data represented as Mean ± SE of mean, (*n* = 3).

Cell viability within GelCryo scaffolds was assessed using the trypan blue exclusion method (Fig. 6). Both 1% and 1.5% GA groups showed >90% viability on Day 1, which remained ≥80% through Day 14. A modest decline was observed by Day 21, though overall viability remained acceptable. These results confirm that neither scaffold formulation exerted cytotoxic effects during the initial two weeks of culture.

Sustained viability over this period suggests that the interconnected porous architecture enabled efficient nutrient and oxygen exchange while limiting accumulation of waste metabolites. The slight reduction at later time points likely reflects increased cell density and diffusion limitations common in 3D cultures. Overall, the data demonstrate that GelCryo provides a cytocompatible microenvironment supportive of long-term cell survival, validating its application in tissue engineering and tumor modeling contexts.

### 4.2. Crosslinking density influences cell proliferation and growth dynamics in GelCryo scaffolds

**Fig. 7:**
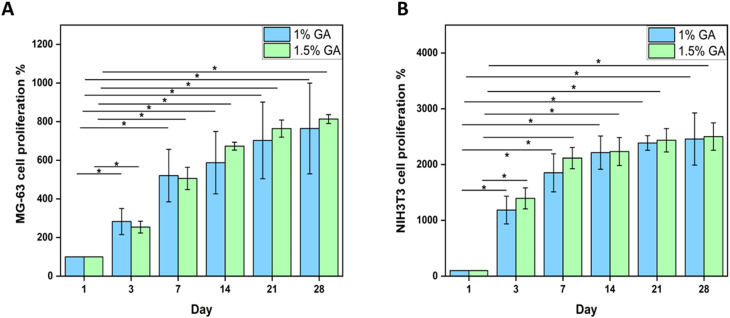
Cell proliferation assay (A) cell proliferation percentage of MG63 (B) cell proliferation % of NIH3T3 cell-seeded scaffolds determined using alamarBlue assay. Statistical significance was tested with one-way ANOVA (*p < 0.05), (*n* = 3).

Quantitative assessment of NIH3T3 and MG63 cell proliferation activity was measured using the Alamar Blue assay. The results, normalized to day 1, confirmed sustained proliferation of NIH3T3 and MG-63 cells within GelCryo scaffolds across 28 days. Both cell types exhibited rapid growth during the first 7 days, followed by a steady plateau, indicating robust early expansion and subsequent stabilisation. Fig. 7A shows the experimental results of MG63 cell proliferation in GelCryo scaffolds, where 1% GA showed rapid rise from negligible day 1 to ∼180% (day 7), ∼300% (day 14), plateauing at ∼350% (days 21–28), while 1.5% GA exhibited superior ∼220% (day 7), ∼400% (day 14–28). The result in Fig. 7B show NIH3T3 in 1% GA GelCryo condition with a negligible start (∼5% at day 1), explosive growth peaking at ∼2000% by day 7, followed by a transient dip to ∼1400% at day 14, and gradual recovery to ∼1900% by day 28, indicating potent but biphasic stimulation. In contrast, 1.5% GA GelCryo exhibits a slightly higher initial response (∼10% at day 1), a similar early peak (∼2100% at day 7), a modest dip (∼1500% at day 14), and stronger sustained proliferation reaching ∼2200% at day 28.

NIH3T3 fibroblasts exhibited ∼15–25% greater maximal proliferation compared to MG63, reflecting their inherently faster doubling time, superior matrix metalloproteinase activity enabling rapid ECM remodeling in soft gelatin environments, and heightened metabolic adaptability in non-osteoinductive 3D matrices lacking hydroxyapatite or bone morphogenetic proteins (BMPs) cues that MG63 preferentially exploits for anchorage-dependent growth. While proliferation trends were similar across formulations, 1.5% GA scaffolds consistently produced marginally higher RFU values than 1% GA scaffolds. Elevated proliferation in 1.5% GA GelCryo (∼10–20% higher across lines) stems from denser crosslinking, which enhances scaffold stiffness, slows proteolytic degradation, and strengthens integrin-mediated cell adhesion to RGD motifs in gelatin, promoting focal contacts and cytoskeletal tension without toxicity at this concentration. This fosters superior nutrient retention and mechanical cues mimicking native ECM, outperforming lower 1% GA, where excessive porosity may dilute bioactivity signals.

**Fig. 8:**
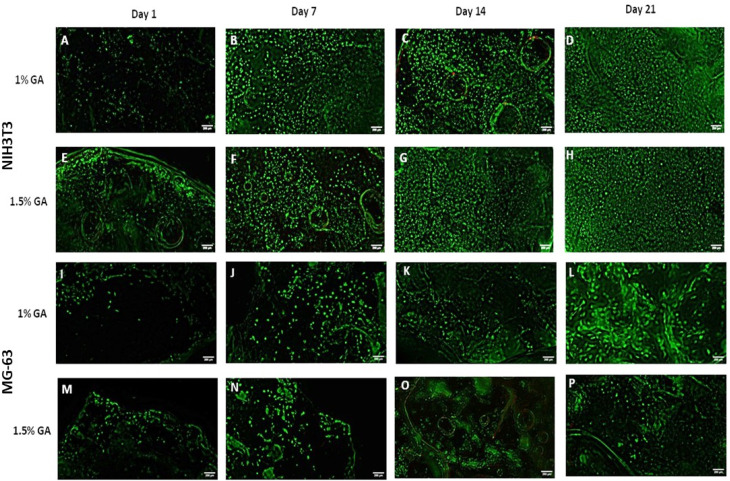
Live/Dead staining of NIH3T3 and MG63 cells after culturing in GelCryo at varying time points. (A–D) MIP of NIH3T3 cells on 1% GA GelCryo on days 1, 7, 14, and 21, respectively (E–H). MIP of NIH3T3 cells on 1.5% GA GelCryo on days 1, 7, 14, and 21, respectively. (I–L) MIP of MG-63 cells on 1% GA GelCryo on days 1, 7, 14, and 21, respectively (M–P). MIP of MG-63 cells on 1.5% GA GelCryo on days 1, 7, 14, and 21, respectively. Magnification 4×, scale bar-200 µm.

Live/dead staining corroborated these findings, with extensive green fluorescence and progressive cell spreading over time (Fig. 8). By day 14, cells had colonised toward the periphery, and overall viability remained high in both scaffold types. OS cells, being of mesenchymal origin, exhibit enhanced proliferative capacity when cultured on scaffolds that recapitulate the architecture of their native extracellular matrix (ECM).^37^ The proliferation study indicates that GelCryo scaffolds closely mimic the bone-like ECM environment, thereby supporting superior cellular growth. The 1.5% GA group displayed greater cell density and spreading, consistent with literature associating moderately stiffer, yet porous matrices with improved proliferation and adhesion.^38^ Collectively, the results establish that GelCryo scaffolds provide a supportive 3D microenvironment for both stromal (NIH3T3) and osteosarcoma (MG-63) cells, with tuneable properties enabling optimisation of proliferative outcomes.

## 5. Drug testing

### 5.1 Physiologically relevant 3D GelCryo models reveal elevated methotrexate IC50 values

**Fig. 9:**
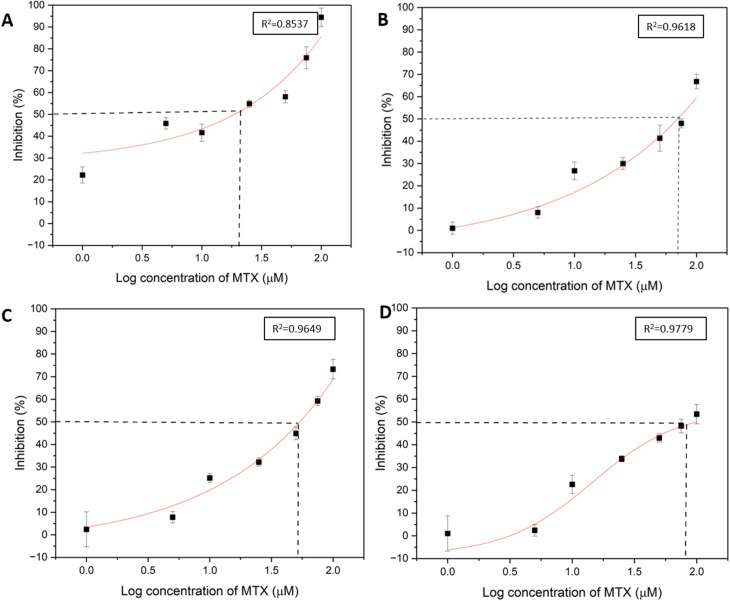
Dose response curve and IC_50_ values of MTX in various cell culture models using 4 parameter non-linear logistic model. Cells were incubated with different doses of MTX for 48 h. (A) 2D cell culture (B) spheroids (C) 3D cell culture in 1% GA GelCryo (D) 3D cell culture in 1.5% GA GelCryo. Cell viability was analyzed using the alamarBlue assay. Data are presented as the mean ± standard deviations. *x*-axis, doses of MTX in µM. *y*-axis, percentage of viability relative to the untreated cells. MTX, methotrexate (*n* = 2).

The cytotoxic response to methotrexate (MTX) was assessed by seeding MG63 cells in 2D monolayers, spheroids (SI, Fig. S2A), and 3D cultures embedded in GelCryo scaffolds at 1% or 1.5% glutaraldehyde (GA) cross-linking (SI, Fig. S2B) following 48 h exposure to MTX concentrations of 0.01–100 µM, with cell viability quantified *via* alamarBlue assay (Fig. 9). Dose–response curves revealed a sigmoidal decline in viability across models, yielding IC_50_ values of 36.2 ± 11.1 µM in 2D cultures, 69.7 ± 11.2 µM in spheroids (intermediate resistance), and markedly elevated values of 53.7 ± 12.9 µM (1% GA GelCryo) and 99.8 ± 22.2 µM (1.5% GA GelCryo), as derived from 4-parameter nonlinear logistic fits (Table 1, Fig. 9, supplementary information, Fig. S3). This progression of increasing IC_50_ from 2D to denser 3D matrices underscores enhanced MTX resistance in physiologically mimetic environments, attributable to impeded drug penetration within crosslinked scaffolds that recapitulate *in vivo* tumour barriers.^39^ Consistent with a study by^40^ which showed that embedding spheroids in PEGDA-GELMA cryogels enhances mechanical stability, supplies ECM-like cues absent in free-floating models, and drives EMT-mediated drug resistance, the MG-63 spheroid model, which partially recapitulates the 3D architecture of tumours demonstrated intermediate drug resistance between 2D and fully embedded 3D cultures.

**Table 1:** Comparison of IC_50_ values in curve fitting models

Cell culture model	4 parameter non-linear logistic fit
2D cell culture	36.2 ± 11.1 µM
3D spheroid culture	69.7 ± 11.2 µM
3D 1% GA GelCryo cell culture	53.7 ± 12.9 µM
3D 1.5% GA GelCryo cell culture	97.8 ± 22.2 µM

A critical outcome of this study is the observed difference in methotrexate (MTX) sensitivity between the two scaffold types. A study conducted by^41^ provides mechanistic validation of the GelCryo MTX resistance trend, showing that increased matrix crosslinking density in hydrogels creates diffusion bottlenecks for small-molecule chemotherapeutics, thereby shifting dose–response curves to the right and elevating IC_50_ values relative to loosely structured or uncrosslinked 3D platforms. The parallel observation of progressively higher IC_50_ (53.7 ± 12.9 µM at 1% GA *vs.* 99.8 ± 22.2 µM at 1.5% GA) in denser GelCryo scaffolds mirrors their findings, confirming that glutaraldehyde-induced network tightening imposes physiological mass transport barriers akin to tumor stroma. This trend aligns with^42^ whose comprehensive analysis establishes that scaffold porosity, pore size, matrix stiffness, and crosslinking density critically govern cellular mechanotransduction, survival signaling, and drug transport barriers in 3D cultures, with denser architectures promoting enhanced chemoresistance through restricted diffusion and ECM-mediated protective responses.Such findings are consistent with literature demonstrating that 3D culture systems with higher matrix density better recapitulate chemoresistance observed *in vivo* compared to 2D cultures.^43^

Co-culturing NIH3T3 fibroblasts with MG63 osteosarcoma cells (SI, Fig. S4) revealed unanticipated reductions in MTX IC_50_ values across all culture formats, with the most pronounced sensitization occurring in denser 1.5% GA GelCryo scaffolds relative to MG63 monocultures. Unlike tumor-derived CAFs that typically confer resistance through IL-6/STAT3 and ECM-mediated protection,^44^this sensitization likely stems from NIH3T3's heightened folylpolyglutamate synthase (FPGS) activity, which rapidly converts MTX to retained polyglutamates—depleting the shared 3D compartment of diffusible monoglutamate available to MG63. The composite alamarBlue readout further amplifies this effect, as NIH3T3's intrinsically lower IC_50_ drives overall metabolic decline. This fibroblast-specific metabolism recapitulates stromal pharmacodynamics absent in monocultures, where matrix barriers alone dictate response. Further FPGS expression and MTX-polyglutamate quantification will clarify the mechanism.

### 5.2 Ki-67/DAPI confirms MTX proliferation arrest at 3D IC_50_ in GelCryo scaffolds

**Fig. 10:**
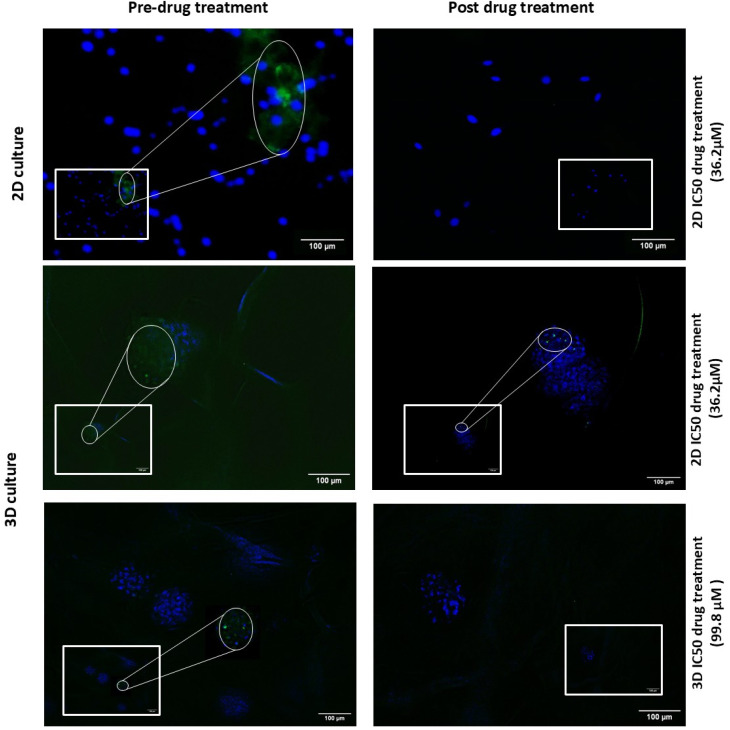
Immunofluorescence staining reveals proliferative capacity of MG63 osteosarcoma cells in gelatin cryogel scaffolds. Representative confocal micrographs showing Ki67 (green) proliferation marker and DAPI (blue) nuclear counterstain in MG63 cells cultured for 7 days within the interconnected pores of the cryogel matrix. Scale bar, 100 µm.

Immunofluorescence analysis of Ki67 (green) and DAPI (blue) staining in MG63 osteosarcoma cells cultured on gelatin cryogel scaffolds for 7 days demonstrated robust proliferative activity, with intense green nuclear labeling of Ki67 in over 70% of DAPI-positive cells prior to drug treatment forming multicellular spheroids within the porous matrix (Fig. 10). The extensive colocalization of Ki67 signal throughout the scaffold depth underscores sustained cell cycle progression prior to the drug treatment, however on exposure to 36.2 ± 11.1 µM of MTX drug concentration, Ki67 signals do not appear, thereby indicating an absence of dividing cells. Untreated 3D GelCryo with 1.5% GA concentration cultures displayed abundant Ki67+/DAPI + multicellular aggregates throughout the scaffold interstices, confirming sustained mitotic activity prior to drug challenge. Post-exposure to 2D-derived IC_50_ MTX, cells retained dense DAPI staining with residual Ki67 foci, indicating incomplete cytostatic efficacy despite nominal IC_50_ attainment. In marked contrast, GelCryo-embedded cells treated at their elevated 3D IC_50_ (99.8 ± 22.2 µM) exhibited near-total Ki67 ablation and sparse DAPI signal, signifying profound proliferation shutdown coupled with widespread cell death—consistent with matrix-amplified drug barriers necessitating higher dosing thresholds for equivalent pharmacodynamic impact. These orthogonal readouts corroborate Alamar Blue-derived IC_50_ hierarchies and underscore GelCryo's fidelity in modeling microenvironmental resistance. This proliferation profile mirrors findings in gelatin-based osteosarcoma models, where 3D architectures foster MG63 aggregation and metabolic resilience, yielding 2-5-fold elevated IC_50_ values against chemotherapeutics compared to 2D cultures due to enhanced cell–cell interactions and diffusion-limited drug penetration.

### 5.3. Higher MTX IC50 in 3D GelCryo cultures is independent of scaffold-mediated drug sequestration

**Fig. 11:**
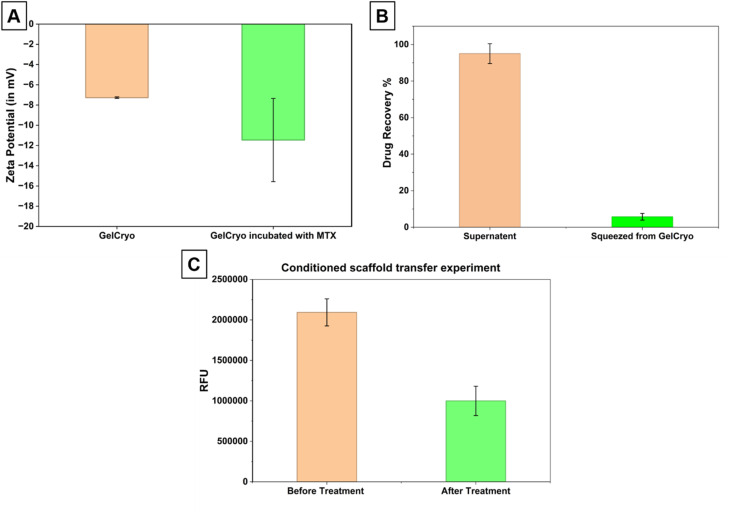
(A) Zeta potential of GelCryo and GelCryo scaffold incubated with MTX. (B) MTX drug recovery percentage from supernatant and after squeezing from the GelCryo scaffold. (C) RFU value of MG-63 cells in 2D before and after treatment with MTX-conditioned media at the 2D IC_50_ drug concentration.

The interaction of MTX *via* surface adsorption or electrostatic interactions can result in a higher IC_50_ value on the GelCryo scaffold compared to 2D cell culture, due to drug retention on the scaffold or inactivation of the drug by scaffold interactions. To study the effect of scaffold-drug interactions, we performed zeta potential analysis to characterise the surface charge of the GelCryo scaffold. GelCryo scaffolds were incubated with MTX for 24 hours to simulate the drug treatment, then crushed and dispersed in PBS (pH 7.0). The zeta potential analysis of GelCryo scaffold exhibited a weak negative potential of −7.27 ± 0.07 mV. After MTX incubation, a slightly negative Zeta potential value (−11.45 ± 4.1 mV) was observed compared to only the GelCryo scaffold (Fig. 11). MTX is negatively charged under neutral pH conditions due to ionisation of the carboxyl groups of MTX.^45^ The zeta measurements suggest that only a modest electrostatic interaction occurs between the GelCryo scaffold and MTX at pH 7.0. To further probe whether non-electrostatic interactions resulted in higher IC_50_ values in 3D cryogels, we performed MTX retention and recovery experiments using the GelCryo scaffold. MTX at 100 µM, the highest concentration used in the study, was incubated with the GelCryo scaffold for 24 hours, replicating the incubation period. After incubation, the GelCryo scaffolds were squeezed, and the supernatant was collected, and the MTX concentration in the supernatant was quantified using UV-vis absorption spectroscopy at 303 nm. The results show that 95.03 ± 5.41% of the MTX drug was recovered from the GelCryo scaffold. These results are in correspondence with the zeta potential study, which suggests minimal interaction of MTX with GelCryo scaffold. To further probe whether drug interaction with the scaffold can inactivate the drug and yield a higher IC_50_ value for the scaffold group compared to 2D, we performed a conditioned scaffold transfer experiment. MTX drug at 36.2 µM, corresponding to the IC_50_ in 2D, was incubated with the GelCryo scaffold for 24 hours. After 24 hours, the conditioned media with the drug were added to MG-63 cells in 2D culture for 48 hours. Prior to 2D cell treatment, cell viability was measured using the Alamar Blue assay, and the same cells were then treated with conditioned media from the GelCryo scaffold. After 48 hours of incubation of conditioned media with the drug, cell viability was again measured using Alamar blue. The result shows a 47.63 ± 6.73% reduction in RFU values compared to prior treatment, suggesting a 47.63% cell viability. These results show that MTX's interaction with the GelCryo scaffold does not affect the drug's activity. Taken together, these findings suggest that the elevated IC_50_ observed in the 3D GelCryo scaffold is unlikely to arise due to drug adsorption, electrostatic interaction or drug inactivation by the scaffold but can be due to the interaction of cells with the GelCryo scaffold, resulting in a higher IC_50_ dose of MTX when MG-63 is cultured in a 3D microenvironment.

## 6. Disscussion

In this study, we explore the potential of GelCryo as scaffolds for 3D culture of osteosarcoma cells. The development of glutaraldehyde-crosslinked gelatin cryogels (GelCryo) addresses a persistent need for physiologically faithful 3D osteosarcoma models that transcend the limitations of 2D monolayers and scaffold-free spheroids, which often underestimate clinical chemoresistance due to absent matrix cues and simplistic geometries. Fabricated with tunable macroporosity (347–678 µm pores, >93% porosity), mechanical robustness (bulk modulus 14–16 MPa), and extended stability under enzymatic challenge, GelCryo scaffolds sustain MG63 osteosarcoma cell viability (>80% at 21 days) and Ki67+ proliferation while imposing realistic diffusion barriers—as evidenced by MTX IC_50_ escalation from 36.2 ± 11.1 µM (2D) and 69.7 ± 11.2 µM (spheroids) to 53.7 ± 12.9 µM (1% GA) and 99.8 ± 22.2 µM (1.5% GA). This density-dependent resistance recapitulates *in vivo* tumour stroma, positioning GelCryo as a versatile *in vitro* platform for both 3D cell culture—fostering ECM-mimetic adhesion, infiltration, and long-term homeostasis—and high-fidelity drug screening, where denser variants (1.5% GA) most accurately forecast poor penetrance akin to patient tumours.

Methotrexate sensitivity was found to be strongly dependent on GelCryo crosslinking density. While cells cultured in 1% GelCryo exhibited an IC_50_ of 53.7 ± 12.9 µM, increasing the crosslinking density to 1.5% resulted in a markedly elevated IC_50_ of 99.8 ± 22.2 µM. Notably, this value exceeded that observed in spheroid cultures (69.7 ± 11.2 µM), indicating that the scaffold provides a more protective microenvironment than cell aggregation alone. The highly crosslinked scaffold possessed a smaller pore size and higher compressive modulus, which likely enhanced cell–matrix interactions and mechanotransduction-mediated survival signaling while promoting a more confined and physiologically relevant microenvironment. Drug recovery studies demonstrated 95% recovery of methotrexate and preservation of its cytotoxic activity, confirming that scaffold-mediated drug sequestration was negligible. Therefore, the increased IC_50_ is primarily attributed to biological adaptations induced by the 3D microenvironment rather than reduced drug availability.

While spheroid cultures are widely employed as 3D tumor models, they primarily recapitulate cell–cell interactions and diffusion gradients. In contrast, scaffold-based models provide an additional matrix component that is particularly relevant for mesenchymal malignancies such as osteosarcoma, where cell–matrix interactions play a central role in regulating proliferation, migration, and therapeutic response. The significantly higher methotrexate IC_50_ observed in the highly crosslinked GelCryo scaffold (96 µM) compared with MG-63 spheroids (69.7 ± 11.2 µM) highlights the contribution of matrix-derived biochemical and biomechanical cues to drug tolerance. These findings suggest that scaffold-based 3D models may more accurately reproduce the osteosarcoma microenvironment and therefore offer improved predictive value for preclinical drug screening.

Ki67 immunostaining revealed extensive proliferative activity within MG63 aggregates cultured in GelCryo scaffolds prior to drug treatment, indicating that cells remained actively cycling despite the elevated IC_50_ observed in 3D culture. Following exposure to the 2D-derived IC_50_ concentration of methotrexate, proliferation was effectively suppressed in monolayer cultures, whereas residual Ki67-positive multicellular clusters persisted within the scaffold. Complete abrogation of proliferative activity was achieved only at the substantially higher 3D IC_50_ concentration, demonstrating that the GelCryo microenvironment confers a drug-tolerant phenotype while maintaining cellular proliferation. Together with the minimal scaffold-mediated drug adsorption observed experimentally, these findings suggest that the increased IC_50_ arises primarily from matrix-driven biological adaptations rather than reduced drug availability. The results further highlight the importance of cell–matrix interactions in regulating chemotherapeutic response in mesenchymal-derived osteosarcoma models and support the use of scaffold-based systems as more physiologically relevant alternatives to conventional spheroid culture.

Such superiority over conventional systems aligns with (Loh and Choong),^42^ who correlated scaffold stiffness/porosity with mechano-transduced survival advantages, and Sandhu *et al.*^39^ documenting crosslinking-induced IC_50_ shifts in hydrogels that mirror GelCryo's transport restrictions. Complementing (Singh *et al.*, ^40^ where cryogel embedding stabilized spheroids and amplified EMT-driven resistance beyond free aggregates, GelCryo uniquely integrates these benefits into a single, autoclavable construct, offering enhanced reproducibility and translational potential for osteosarcoma pharmacodynamics. Compared with synthetic polymer cryogels, which typically provide superior batch reproducibility and chemical uniformity, GelCryo offer a more biomimetic microenvironment because they retain collagen-derived cell-adhesive motifs and support enzyme-responsive remodeling. Although variability in gelatin source may affect scaffold-to-scaffold consistency, this concern can be minimized during scale-up by using a single well-characterized gelatin grade with predefined physicochemical specifications, performing incoming batch qualification, and maintaining strict control over crosslinker concentration, polymer content, and cryogelation conditions.

GelCryo's injectability was demonstrated by its rapid shape recovery under syringe extrusion (SI, Video S1), which is attributed to the presence of imine bonds between amine groups of gelatins and crosslinker aldehydes maintain network integrity and enables >95% volumetric recovery during compression–decompression cycles. The restored 3D scaffold immediately regains its original storage modulus, creating a permissive microenvironment for osteosarcoma cell proliferation in drug screening assays.^36^ This injectability-profile-positioned GelCryo as a promising candidate for *in situ* cancer model establishment and localized drug delivery applications. Coupled with sigmoidal degradation (89 h at physiological collagenase for 1.5% GA) and robust protein retention (>300 µg cm^−3^), these traits enable controlled elution akin to established cryogel carriers, addressing osteosarcoma's intratumoral penetration deficits where conventional dosing falls short.

However, future investigations should prioritize comprehensive validation of the GelCryo 3D platform's capacity to predict *in vivo* chemotherapeutic responses. Expanding testing to mechanistically diverse agents beyond MTX—including anthracyclines (doxorubicin), platinum compounds (cisplatin), and molecularly targeted inhibitors—across chronic exposure regimens would clarify whether the observed resistance profile represents a MTX-specific adaptation or a generalized tumor-stromal chemotolerant state mechanistic dissection requires parallel assessment of apoptotic induction (caspase-3/7 activity, TUNEL), cell cycle perturbations (flow cytometry), hypoxic fraction (pimonidazole/EF5 immunofluorescence), and transcriptomic/proteomic signatures of resistance pathways (*e.g.*, ABC transporters, anti-apoptotic Bcl-2 family, EMT markers). While Matrigel remains a widely used matrix for 3D spheroid and organoid culture due to its rich biological composition, its undefined formulation, substantial lot-to-lot variability, and limited mechanical customization restrict its utility in standardized drug screening studies. GelCryo, by comparison, provides a precisely tunable and structurally robust alternative, combining interconnected porosity with adjustable stiffness and degradability to better support long-term culture and controlled mass transport. Matrigel are used to make spheroids that replicate cell–cell interactions and to study their drug resistance. In contrast GelCryo replicate cell–matrix interactions especially important for cell-derived from mesenchymal origin like osteosarcoma and potentially can be used to study drug resistance due to cell–matrix interactions. Further by incorporating patient-derived osteosarcoma cells or heterotypic co-cultures incorporating fibroblasts, endothelial cells, and macrophages within GelCryo matrices further replicate complex tumor micro-environment. Collectively, these future developments may establish scaffold-based osteosarcoma models as a more predictive platform than conventional spheroid cultures for investigating tumor biology and evaluating anticancer therapeutics in mesenchymal-derived malignancies.

## 7. Conclusion

This study demonstrates that GelCryo scaffolds crosslinked with 1% and 1.5% GA provide biocompatible 3D platforms capable of sustaining osteosarcoma and fibroblast growth. While both variants supported high viability and proliferation, the 1.5% GA formulation exhibited superior physicochemical attributes, including greater mechanical strength, reduced swelling, smaller interconnected pores, and enhanced enzymatic stability. These features created a denser, more restrictive microenvironment that supported higher baseline metabolic activity and increased methotrexate resistance, thereby better recapitulating the chemo resistant tumor niche. The marked differences in drug response between 2D and 3D cultures underscore the limitations of conventional assays and highlight the translational relevance of matrix-tunable scaffolds. Overall, the 1.5% GA GelCryo emerges as a predictive *in vitro* model for anti-cancer drug screening in osteosarcoma, offering a physiologically relevant alternative to traditional systems.

## Conflicts of interest

There are no conflicts to declare.

## Supplementary Material

RA-016-D6RA03165J-s001

RA-016-D6RA03165J-s002

## Data Availability

All data for this communications article was created by Mathew Peter, using the facilities in the Manipal Institute of Technology, MAHE. The data supporting this article is included in the main text as well as in the supplementary information (SI). Supplementary information is available. See DOI: https://doi.org/10.1039/d6ra03165j.
