# Supplementary material for: Development of gelatin based cryogel model for drug screening application in osteosarcoma
Source: RSC Adv. 2026 Jul 3;16(35):35763–79. doi: 10.1039/d6ra03165j (PMC13329851; doi:10.1039/d6ra03165j)
Supplement: RA-016-D6RA03165J-s001 [file RA-016-D6RA03165J-s001.pdf]

Supplementary Information (SI) for RSC Advances.
This journal is © The Royal Society of Chemistry 2026

Supplementary Materials

Development of Gelatin based Cryogel Model for Drug Screening Application in Osteosarcoma

Ponnamma Mandeda Madaiah[1],Rudra Nath Ghosh[2], Mathew Peter[2*]

[1]Manipal School of Life Sciences, Manipal Academy of Higher Education, Manipal, 576104, India.

[2]Department of Biomedical Engineering, Manipal Institute of Technology, Manipal Academy of Higher Education, Manipal, 576104, India.

* *Corresponding Author:* *mathew.peter@manipal.edu*

Injectability of Cryogel

Injectability og cryogel.mp4

Video S1: 1.5% GA GelCryo disc (8 mm) is deformed through a 16G needle and rapidly recovers >95% original morphology within 10 s upon PBS deposition, demonstrating shape-memory and macroporosity preservation for injectable drug delivery.

Optimization of GelCryo

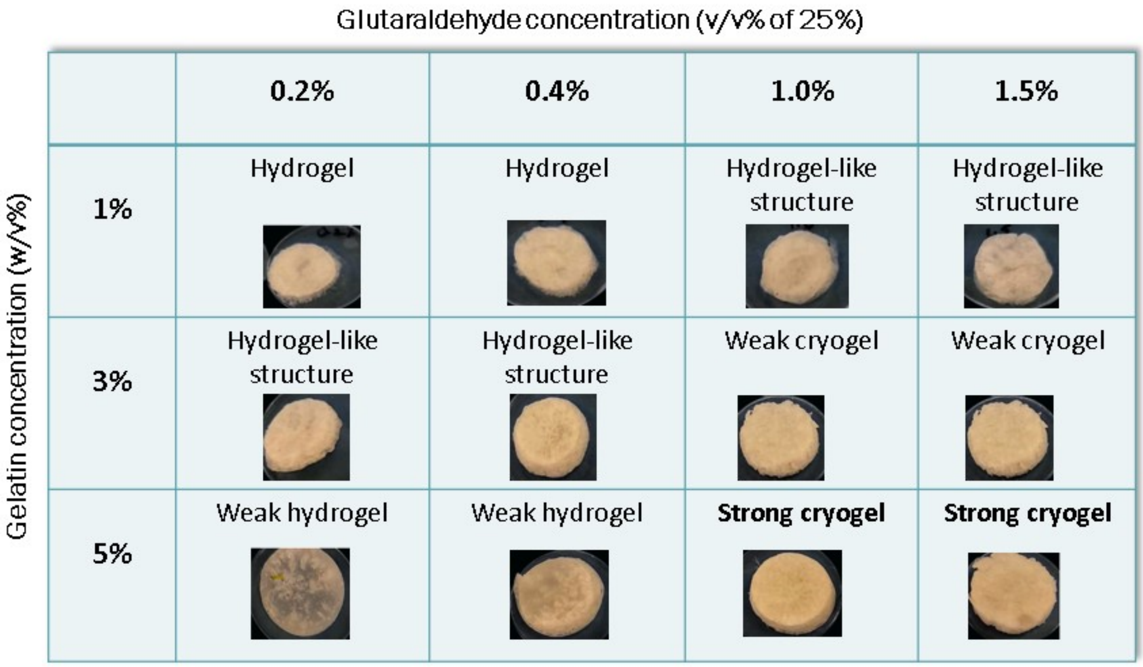

Figure S1: Representative image of different cryogels fabricated with different concentrations of gelatin and glutaraldehyde.

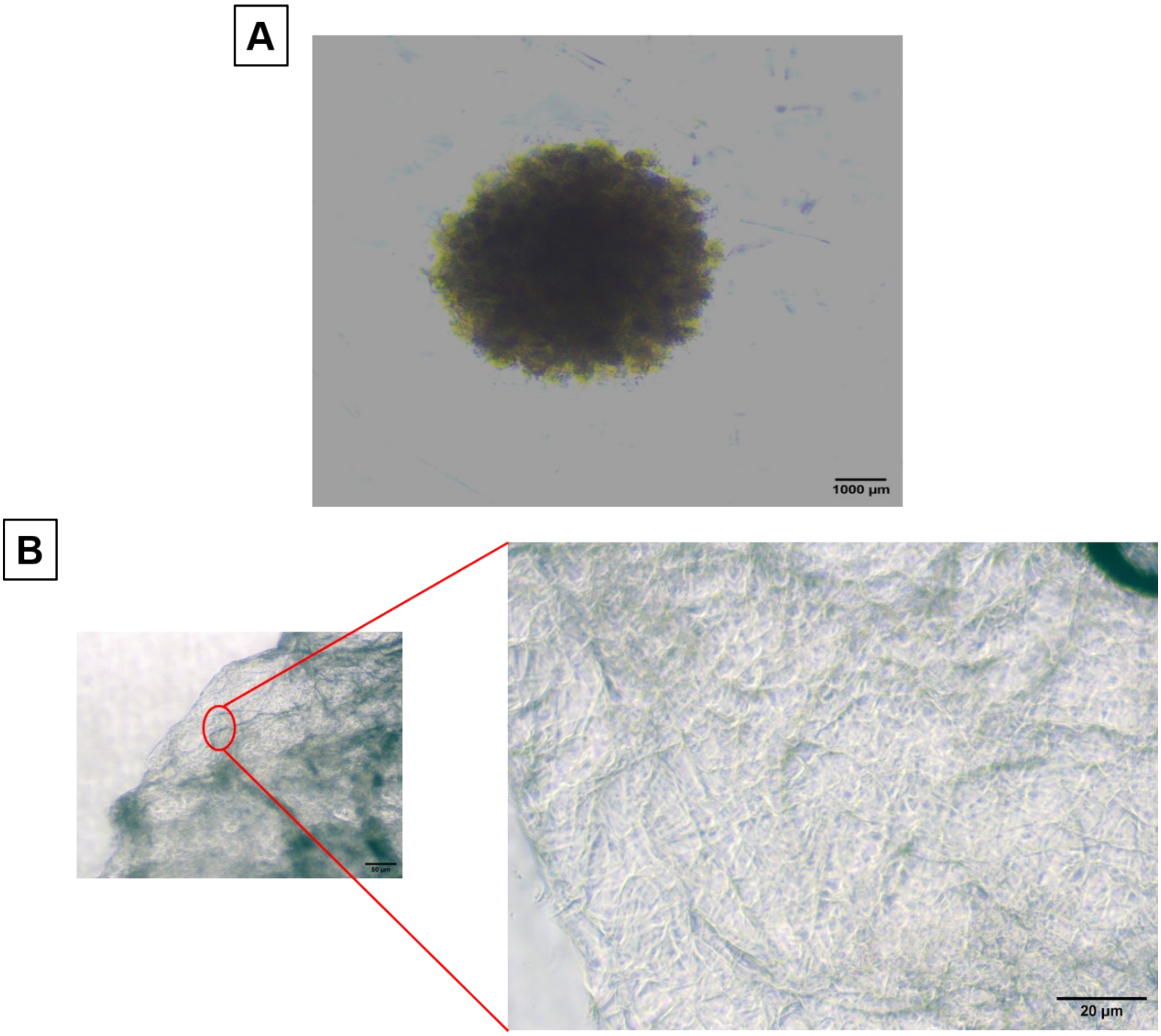

Figure S2: Phase contrast images of MG-63 osteosarcoma cells in (A) 3D spheroid and (B) Seeded in GelCryo scaffold

Drug Response Study

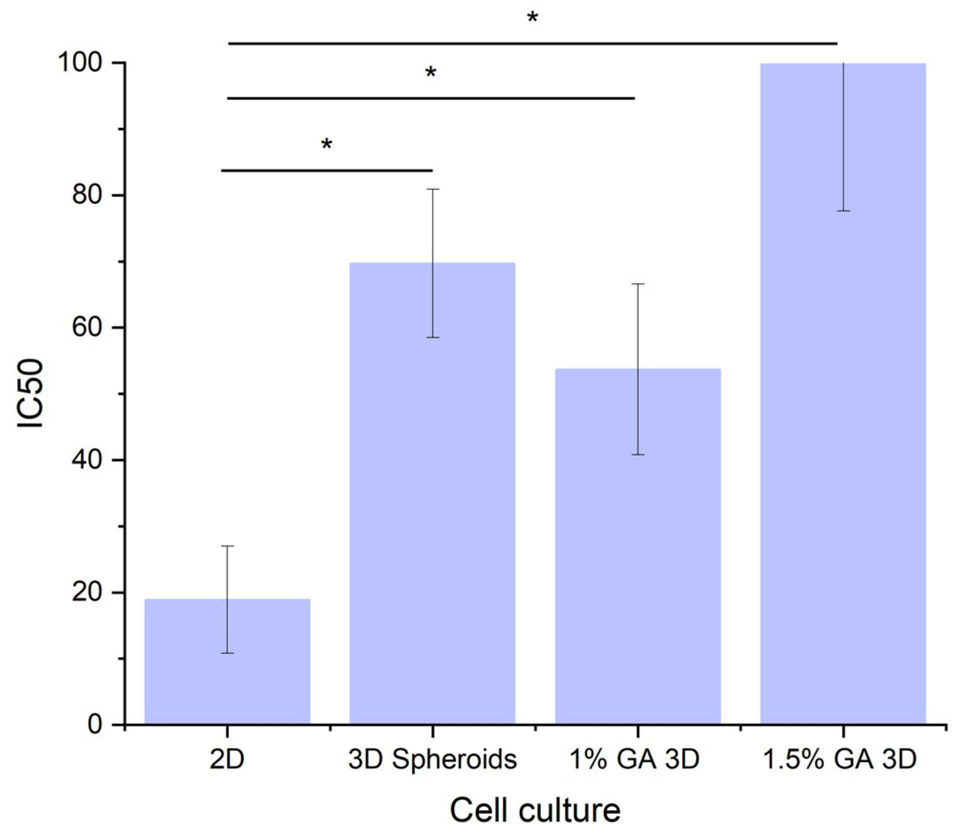

Figure S3: **Comparative MTX IC50 values in MG63 osteosarcoma cultures.**
IC50 concentrations (µM) of methotrexate after 48 h treatment, derived from sigmoidal dose-response curves via 4-parameter nonlinear regression in 2D monolayers, 3D spheroids, 1% GA GelCryo, and 1.5% GA GelCryo. Data shown as mean ± SD (n=2); *$p<0.05$ vs. 2D (one-way ANOVA with post-hoc Tukey).

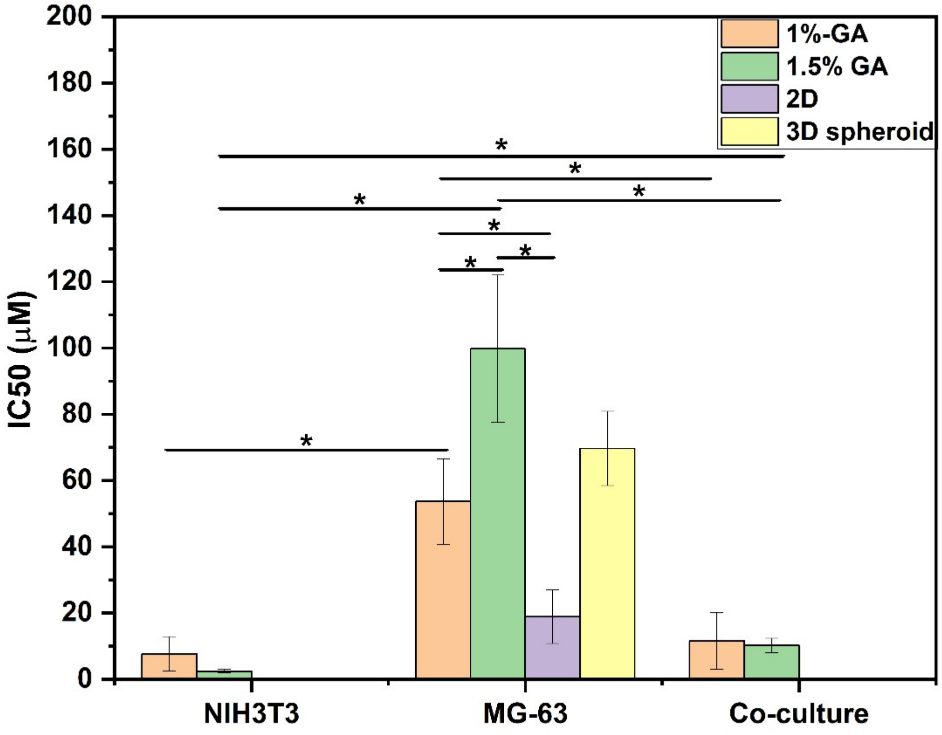

Figure S4: MTX IC50 values across cell types and culture conditions. Bar graph depicting half-maximal inhibitory concentrations (IC50, μM) of methotrexate determined by 4-parameter logistic fitting following 48 h exposure. NIH3T3 fibroblasts, MG63 osteosarcoma cells, and MG63:NIH3T3 co-cultures were evaluated in 2D monolayers (purple), 1% GA GelCryo (orange), 1.5% GA GelCryo (yellow), and spheroids (purple). Data represent mean ± SD (n=2–3); statistical significance by one-way ANOVA (*p<0.05 vs. 2D). Co-culture and denser scaffolds exhibit pronounced resistance, consistent with stromal modulation and matrix barriers.
